# X-Rays in Medicine and Surgery

**Published:** 1898-02

**Authors:** 


### X-Rays in Medicine and Surgery.

Raw (*Lancet*, No. 3,821), experiments on the cadaver, foreign bodies being placed within. All of these show distinctly, and in all their parts in the skiagraph. The outlines of the viscera are well delineated also, the skeleton being most distinct. In another cadaver thick copper wire was placed around the edges of the heart, lungs, liver, spleen and kidneys. Four calculi were placed in the gall bladder, and calculi in the renal cortex, in the pelvis and in the ureter. The cavities were carefully closed and ribs replaced. With an exposure of sixty minutes a good negative was secured which showed all the points, the calculi being especially clear.

